# Notes on Laminaria Tents

**Published:** 1871-05

**Authors:** J. C. Nott

**Affiliations:** New York


### *Notes on Laminaria Tents.* By J. C. Nott, M. D., New York.

[Amer. Jour. Obstet.]

Much diversity of opinion exists among gynæcologists as to the comparative merits of sponge and laminaria tents. It is conceded generally that sponge-tents should be used with caution, as they are occasionally followed by metritis or inflammation of tissues connected with the uterus—the sponge, in expanding,

*Traite des Maladies Inflammatoires du Cerveau. Two Vols., Paris, 1859.

drives its little projections into the substance of the mucous lining, which is abraded or lacerated in the act of removal, causing more or less bleeding, and often a good deal of pain. Another objection to the sponge is, that after absorbing the animal fluids, it becomes excessively putrid and offensive, and, there is good reason to believe, may produce blood-poisoning. These objections to sponge hold, to say the least, in a much less degree against the laminaria digitata. When the uterus is irritable—sensible to the probe or touch of the finger—when there is any tenderness or disease of surrounding tissues, it is a good rule to dispense with the use of tents of any kind, and the violation of this rule often leads to serious complications.

Our instrument makers and druggists import the laminaria both in the raw state and made up into tents ready for use. It comes from France, England, California, and the Bay of Fundy, on our Atlantic coast. I have not been able, so far, to obtain satisfactory information as to the natural history of the article, or the precise localities from which the specimens are taken. I have made a series of experiments on the varieties found in our market, which go to show that they differ much in their physical characteristics, and their applicability to the purposes for which they are employed.

From Messrs. Tiemann & Co., 63 Chatham street, I obtained specimens of the raw material from California in its rough state. These differ much in appearance from those of other localities; they are generally hollow, of a dirty, pipe-clay color, covered with rough bark, cylindrical, and as large as lead-pencils of different sizes. When placed in water for twenty-four hours, they absorb and expand very slowly, and, after fully expanding, remain so hard, woody, and uneven, as to make one believe that they would bruise and irritate the cervix uteri. I should, therefore, condemn the California laminaria as unfit for use. It may be that the specimens I have seen are *too old*, and that younger plants would answer better. I am told, too, that there is a difference in the male and female plants—the female being hollow, and the male solid. That I have seen from California is hollow. I hope to get other specimens from the Pacific coast for experiment.

From Mr. Hornstein, No. 52 Maiden Lane, I procured some specimens of the crude laminaria, which he had imported from France, and from which he manufactures very pretty-looking tents for this market. This material resembles closely that of California in physical qualities. It expands slowly in water, and, when fully saturated, is hard, uneven, and woody in texture—almost as hard as a piece of white pine wood—and therefore objectionable.

Judging by the eye alone, the laminaria tents, as presented for sale by our surgical instrument makers, are of two kinds, and very different in appearance. The first are beautifully finished, perfectly smooth and cylindrical, with nicely-rounded extremity of tawny, yellowish, or greenish color; and are entirely denuded of bark. From their roundness, smoothness, and bougie-like shape, they look very tempting; but, so far as I have tested them, they are objectionable—they expand too slowly; after being soaked in water for twenty-four hours, they still feel hard and woody, and many of them, when fully expanded by moisture, are almost triangular in shape, presenting edges as sharp as those of a three-cornered file, and well calculated to do injury to the uterus. These tents differ a good deal among themselves—those of tawny color preserve pretty well, when expanded, their regular cylindrical shape, while the greenish ones develop into irregular, angular shapes. Both, I think, should be rejected on account of their hardness and slowness of expansion. Tents of such hardness and tardiness of expansion are likely to irritate, provoke uterine contractions, and be expelled. The second kind of prepared laminaria tents above alluded to, as sold in our market, though by far the best, are not, I think, generally fully appreciated by the profession on account of their rough, ungainly appearance, and from their usual smaller size. This variety has been obtained by Messrs. Tiemann & Co., from the Bay of Fundy, and is peculiar in appearance; it varies in size from a knitting-needle to that of a large goose-quill; its shape is ovoidal near the root, and the stem, which is from one to two feet in length, becomes as flat as tape towards the upper extremity. It is covered by a thin cuticle, which is as black as charcoal, and is more or less rough from the unevenness of the cuticle. When prepared as tents for sale, it is cut into pieces of proper length, pared round at the extremity, and the surface is scraped off a little, to remove the unevenness, but enough of the bark remains to preserve their black color. These tents, made from the Bay of Fundy laminaria, absorb moisture rapidly, and will expand to their maximum in less than half the time required by the European or California varieties. They soon, when placed in water, become as soft and pliable as the tendon of a muscle while fresh. These tents, as sold, look so rough and hard, so like a splinter of wood, that I had a great prejudice against them until I learned how to manage them—I did not see how the uterus could tolerate them.

The manner of using them is simply this: Take one of these tents, no matter how rough or rude in shape, and immerse it in hot water a few minutes, when it becomes coated with mucilage like slippery-elm bark, and so flexible that it may be shaped in any curve to suit the channel of the uterus; it is then as easily introduced as a well-oiled gum-elastic bougie of the same size. The patient is unconscious of its passage or presence; and if a single one is not sufficient to insure the amount of dilatation desired, two, three, or more, may be inserted and placed beside each other. These may be allowed to remain twelve to twenty-four hours, produce no irritation as do the sponge-tent, do not become putrid; and, from their smoothness and mucilaginous coating, are easily removed without producing any abrasion of the cervical canal. These tents can be readily glided into narrow canals, where the sponge could not pass, and are particularly valuable in commencing the dilatation of a very narrow canal. To guard against their expulsion, I frequently place over the os uteri a pledget of cotton wet with persulphate of iron; the vagina contracts around it, from its astringency, and holds the tents in place.

There is another fact, and perhaps one of importance, that I have not seen alluded to by any writer—viz: *the facility with which laminaria tents may be medicated. Watery* solutions of iodine, morphine, and other articles, are absorbed by them very readily, and the substance dissolved is retained after the tent is again dried. I immersed a moderate-sized Bay of Fundy tent in Magendie's solution of morphine for twenty-four hours, and ascertained at the end of this time that it had *absorbed about* ʒij. *of the solution*, containing four grains of morphia. I found, also, that if the tent be well softened in water, and, when still soft and moist, a grain of dry morphia be placed upon the extremity, it will absorb it in a few minutes, and retain it when dried. A tent medicated in this way, introduced into the uterus, will soon produce the constitutional effects of morphia. In using tents, might it not be well, in many cases, to medicate them with morphia, both for the constitutional and local effects.

Some months ago, I medicated some tents with morphia, laid them aside, and carelessly got them mixed up with others that had not been medicated. About a month ago, I introduced a tent, which I supposed to be unmedicated, into the uterus of a lady. In about four hours afterwards, she sent for me, to say she was drugged with morphia. I declared my innocence, but she insisted on being under the influence of morphia, and said that, the few times she had taken it, the same disagreeable symptoms had been produced. On investigating the matter fully, I found that I had inadvertently used one of the tents medicated with morphia. After removing the tent, I washed it thoroughly with warm water, and, touching it to my tongue, the bitter taste of the morphia was still strong. Tents soaked twenty-four hours in Lugol's solution of iodine imbibe it freely, and, after they are dried, if placed in water, give it out again quickly; the water assumes the iodine tint in a few minutes. I have also tried carbolic acid dissolved in very dilute alcohol, but the carbolic acid, being volatile, might not be retained any great length of time. Most of the salts soluble in water, I presume, would be readily absorbed. The sea-tangle will not absorb glycerine or alcohol, and cannot, therefore, be medicated by immersion in solutions made of these fluids.

I have not carried my experiments far enough yet, in the practical application of medicated laminaria tents, to say what their true value may be; but they absorb salts so freely, retain them indefinitely, and give them out so readily, that I cannot help believing they may be made useful. Sponge-tents have certainly been medicated to advantage.

My experiments thus far are not complete, and I throw these remarks out more as hints, hoping that others may test the facts more fully.

The following are the conclusions I should at present draw from my observations:

1st. Where moderate dilatation is required, the laminaria is preferable to the sponge-tents.

2d. If placed in warm water, just before introduction, for a few minutes, they become flexible, coated with mucilage, are easily curved to suit the cervical canal, and may be inserted with the utmost facility.

3d. From their smoothness and softness, they are removed without force, and produce no abrasion or irritation.

4th. They may be medicated with morphia, iodine, or anything *soluble in water*, but do not absorb alcoholic solutions or glycerine. After being so charged, they may be dried and kept for use an indefinite time.

5th. They do not become putrid, and therefore poisonous, as do sponge-tents, and may therefore be retained twenty-four hours or more with impunity.

6th. The black, ovoid laminaria, from the Bay of Fundy, is much preferable to the other varieties yet brought to our markets, and free from the objections I have seen made to laminaria by some writers.

7th. The laminaria will be found of great benefit in obstructive dysmenorrhœa, if introduced a few days before the menstrual period, and also in cases of uterine catarrh connected with contracted cervix; they prepare the way well, too, for all intra-uterine medication. In either case, if softened in hot water before introduction, they rarely produce any pain or irritation.

8th. I think it better to insert several small tents than one large one, as the small ones expand more rapidly than the large ones.

